# Composition-Dependent Protein–Material Interaction of Poly(Methyl Methacrylate-*co*-styrene) Nanoparticle Series

**DOI:** 10.3390/ijms242216390

**Published:** 2023-11-16

**Authors:** Barbara Seifert, Stefan Baudis, Christian Wischke

**Affiliations:** Institute of Active Polymers, Helmholtz-Zentrum Hereon, 14513 Teltow, Germany

**Keywords:** nanoparticles, protein interaction, poly(methyl methacrylate-*co*-styrene), carrier systems

## Abstract

Polymer nanoparticles continue to be of high interest in life science applications. Still, adsorption processes occurring in protein-containing media and their implications for biological responses are not generally predictable. Here, the effect of nanoparticle composition on the adsorption of bovine serum albumin (BSA), fibronectin (FN) and immunoglobulin G (IgG) as structurally and functionally different model proteins was explored by systematically altering the composition of poly(methyl methacrylate-*co*-styrene) nanoparticles with sizes in a range of about 550 nm. As determined by protein depletion from the suspension medium via a colorimetric assay, BSA and IgG adsorbed at similar quantities, while FN reached larger masses of adsorbed protein (up to 0.4 ± 0.06 µg·cm^−2^ BSA, 0.42 ± 0.09 µg·cm^−2^ IgG, 0.72 ± 0.04 µg·cm^−2^ FN). A higher content of styrene as the more hydrophobic polymer component enhanced protein binding, which suggests a contribution of hydrophobic interactions despite the particles exhibiting strongly negatively charged surfaces with zeta potentials of −44 to −52 mV. The quantities of adsorbed proteins were estimated to correspond to a confluent surface coverage. Overall, this study illustrated how protein binding can be controlled by systematically varying the nanoparticle bulk composition and may serve as a basis for establishing interfaces with a targeted level of protein retention and/or presentation.

## 1. Introduction

Nanoparticles expose large specific surfaces to their surrounding medium, such as biological fluid, and are thereby subject to protein adsorption. Often, protein adsorption to polymer surfaces is considered to be undesired, and, typically, surface modifications are applied to prevent protein adsorption [1]. In some cases, however, an advantageous adsorption of specific molecules has been reported, which may subsequently mediate surprising nanoparticle functions such as brain uptake via apolipoprotein E accumulated from the serum at the particle surface [2]. Despite the relevance of protein adsorption being well accepted, there are few systematic studies that explore a model nanoparticle series with systematic alteration of the composition of the matrix polymer and its impact on protein adsorption [3]. Instead, most studies were conducted with model nanoparticles, such as from pure polystyrene with different surface modifications, typically by pre-adsorption of surfactants [4,5,6,7,8,9]. Furthermore, etching of the surfaces of polystyrene towards an altered topography was suggested to affect the quantities of adsorbed proteins [10].

Against this background, it would be of interest to understand whether the bulk composition of nanoparticles rather than just surface modifications can be relevant for protein adsorption. Apparently, substantial changes of material characteristics such as hydrophobicity are known to correlate with protein adsorption [1]. For instance, when preparing nanoparticles from poly(D,L-lactide-*co*-glycolide) (PLGA) and, alternatively, from its diblock copolymer with poly(ethylene glycol) (PEG-PLGA), a lower protein adsorption was found for PEG-PLGA, presumably due to hydrophilic PEG-rich domains being present at the particle surface [11]. Furthermore, despite similar coverage of PLGA particles with surfactant, more hydrophilic PLGA with free carboxyl groups adsorbed almost tenfold more protein than more hydrophobic ethyl end-capped PLGA, suggesting that ionic interactions might dominate over hydrophobic interactions in certain cases [12]. In addition to those observations with individual materials, it would be of interest to realize polymer-composition-dependent control of protein adsorption processes by a series of materials with tunable composition and thereby tunable protein adsorption. Such systems, which may allow one to support, prevent or specifically direct the protein adsorption pattern, could later be useful as they affect subsequent processes such as cellular recognition of nanoparticles, e.g., as carriers in medicine and biotechnology or in bioanalytics.

Considering polystyrene as the most common model material for nanoparticles in mechanistic protein adsorption studies, it was here explored whether introducing methyl methacrylate (MMA) as a more hydrophilic comonomer would allow control of protein adsorption without the need to conduct complex surface chemistry as additional steps after particle preparation. Model studies with thin films indicated substantially different protein interactions with polymer segments from these comonomers [13]. Accordingly, the molar concentrations of MMA and styrene (Sty) were systematically altered when synthesizing poly(methyl methacrylate-*co*-styrene) nanoparticles, here abbreviated as P(MMA-Sty)x, with x representing the molar content of Sty. In order to allow simultaneous preparation of nanoparticles with different compositions, a synthesis robot was applied for parallel direct synthesis of the nanoparticles by soap-free emulsion polymerization [14]. The properties of the particles and the adsorption of a number of different model proteins were studied in order to allow conclusions on whether P(MMA-Sty) may enable a tuning of the amount of adsorbed protein and/or mediate a preferential adsorption of specific proteins.

## 2. Results

**Figure 1:**
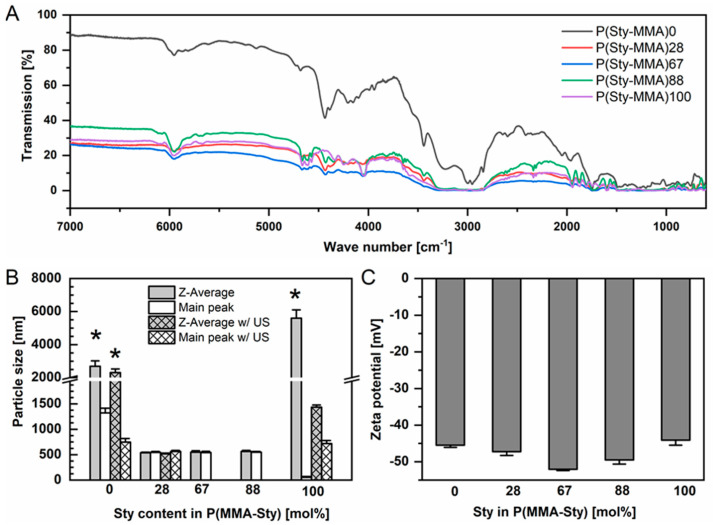
Nanoparticle characterization by (**A**) FTIR spectroscopy via DRIFT technology for determining compositions by multivariate analysis [15], (**B**) dynamic light scattering for determination of mean particle size (n = 3, mean, S.D.) and (**C**) zeta potential analysis (n = 3, mean, S.D.). The particle sizes were determined without or with additional application of ultrasound for redispersion (ultrasonic bath, 5 min). Samples indicated with * are expected to be aggregated, i.e., individual particle sizes are smaller than determined by the light scattering method.

A series of P(MMA-Sty) nanoparticles was synthesized by soap-free emulsion polymerization by robot-assisted synthesis [14], and they were purified by extensive dialysis to remove unreacted monomers. FTIR analysis in the DRIFT mode confirmed that a systematic variation of particle matrix composition between 0 and 100 mol% Sty was achieved. Exemplary FTIR spectra are depicted in Figure 1A which show a typical resolution for DRIFT measurements and differences in the maximum transmission as linked to powder packing. Nevertheless, according to established methodologies based on multivariate data analysis [15], the composition of the particles could be characterized. The determined Sty content was used in sample labels, e.g., P(MMA-Sty)88.

The synthesis of lattices from such water-based emulsion polymerization is often followed by downstream processes to collect the polymer by triggering particle aggregation, subsequent filtration and, finally, polymer drying. In contrast, here, the synthesized product was further used as nanoparticles in aqueous dispersion, i.e., a stabilized colloidal state was anticipated. The analysis of nanoparticles by dynamic light scattering (DLS) revealed homogeneous particle sizes for P(MMA-Sty)28, P(MMA-Sty)67 and P(MMA-Sty)88 in a range of 550 nm. In contrast, for P(MMA-Sty)0 and P(MMA-Sty)100 synthesized only from MMA and Sty, respectively, first DLS analysis suggested the presence of some microparticles with an apparent broad size distribution (Figure 1B). Upon sonication, smaller particle sizes were detected, which were still larger than those of materials from MMA/Sty mixtures (copolymer particles) and exhibited a high polydispersity index (Table 1).

**Figure 2:**
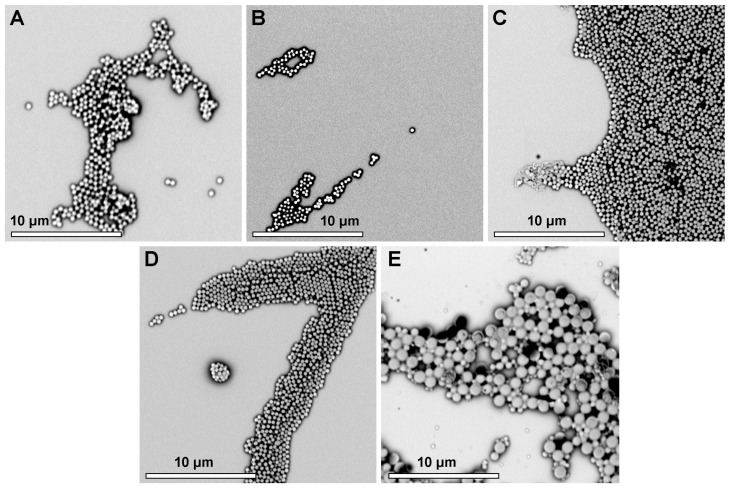
Scanning electron microscopy of nanoparticles for (**A**) P(MMA-Sty)0, (**B**) P(MMA-Sty)28, (**C**) P(MMA-Sty)67, (**D**) P(MMA-Sty)88 and (**E**) P(MMA-Sty)100.

In order to understand whether the DLS data for sonicated samples corresponded to fragments of particle created by sonication, disaggregated primary particles or remaining agglomerates that could not be easily dispersed by ultrasound, the size and morphology of samples were additionally studied by scanning electron microscopy (SEM) (Figure 2). Importantly, this investigation confirmed the presence of homogeneously sized nanoparticles for P(MMA-Sty)0, suggesting that the DLS measurements displayed artefacts by nanoparticle aggregation. In contrast, P(MMA-Sty)100 was shown to contain much larger particles and a broad size distribution, corresponding well to the DLS measurements with sonication. These differences in particle size distribution with variation of the comonomer feed may be a consequence of phase-distribution-related phenomena during nanoparticle synthesis.

Protein–material interaction cannot be assigned to a single physicochemical parameter but is a highly complex process. Obviously, analyzing nanoparticle surface properties is required where particle charge is believed to be a relevant parameter affecting protein binding [1,16]. The zeta potential can be employed as a measure of surface charge and was determined in a low-conductivity medium (~50 µS·cm^−1^), i.e., at conditions where it was close to the Stern potential. Considering the initiator employed for nanoparticle synthesis, ammonium persulfate, sulfate moieties decorate one end of each polymer chain. These groups are expected to be exposed at the particle surface facing the aqueous medium, which justifies the determined clearly negative charges with zeta potentials varying between −44 and −52 mV in aqueous medium (Figure 1C). No systematic effect of composition on particle charges was observed. This can be justified as particle charge is defined in all cases by the negatively charged headgroup of the polymer chains rather than its backbone composition (ratio of repeat units).

In this study, the biointeraction of such nanoparticles was studied with single proteins in order to avoid competing adsorption effects. The following model proteins were selected: bovine serum albumin (BSA) as the most frequent protein in blood plasma, a dysopsonin and a common reference standard for adsorption studies; immunoglobulin G (IgG) as a major opsonin and important protein in the humoral immune response; fibronectin (FN) as an important adhesion-mediating protein in cell biology and, in its insoluble form, part of the extracellular matrix. Another aspect in choosing these model proteins was the increasing molecular weight ranging from ~66 kDa for BSA and ~150 kDa for IgG to ~450 kDa for FN.

**Figure 3:**
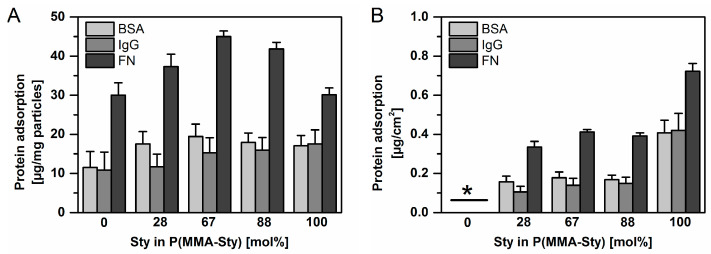
Single protein adsorption studies for different model proteins. (**A**) Data analysis related to the mass of employed nanoparticles. (**B**) Data analysis related to surface areas of the investigated particles (* particle aggregation for P(MMA-Sty)0, data presentation not possible). Protein binding of bovine serum albumin (BSA), human immunoglobulin G (IgG) and bovine fibronectin (FN) was determined by protein depletion from the suspension medium with 1 mg·ml^−1^ nanoparticles and 50 µg ml^−1^ of the respective protein (n ≥ 6; mean, S.D.).

The selected proteins have very different concentrations in physiological fluids such as serum, ranging over at least two orders of magnitude. For a standardized setup, here, concentrations of 50 µg ml^−1^ were employed for all proteins. In order to exclude kinetic effects, an incubation period of 24 h was selected, which should correspond to an equilibrated condition of binding, spatial rearrangement and detachment processes. Depending on the protein and particle type, typically 25–85% of the protein was depleted upon incubation with 1 mg·ml^−1^ nanoparticles. Albumin and IgG showed a trend towards a slightly higher protein adsorption with increasing Sty content when referring to the mass of protein adsorbed on a specific mass of nanoparticles (Figure 3A). Generally, the adsorbed quantities of FN were substantially higher than for BSA and IgG, which may be explained by their physiological function of nonspecific binding to foreign surfaces. When again relating adsorbed quantities to the mass of employed nanoparticles, a hyperbolic correlation of FN adsorption and nanoparticle composition with highest adsorption for medium Sty content was detected (Figure 3A).

Since the sizes of nanoparticles differed in some cases (Table 1), the quantities of adsorbed protein were also related to the surface area exposed by the different types of nanoparticles. It should be noted that for P(MMA-Sty)0, the entire surface of each primary particle may not be effective for interaction with proteins due to nanoparticle aggregation as described above. Accordingly, the average surface areas (Table 1) calculated for each particle composition based on the DLS data may possibly underestimate the accessible surface for P(MMA-Sty)0 as they do not include interparticulate space that may partially be penetrated by the dispersion medium. Therefore, the protein adsorption presented in Figure 3B in relation to the nanoparticle surface area does not include data for P(MMA-Sty)0. However, for all other compositions, a trend towards higher protein adsorption with increasing Sty content was revealed for all studied proteins.

The interaction of proteins with interfaces can lead to different levels of surface coverage. In principle, proteins may form islets of bound proteins, a confluent monolayer or—once adsorbed—may promote the binding of further layers of protein. Previously, for polystyrene nanoparticles prepared by a different polymerization technique, a surface coverage of ~50% was reported for BSA at pH 6 when relating the number of bound molecules to their space requirements estimated by their cross-sectional area [17]. In order to conclude whether the levels of protein binding observed here were in the range of either of the aforementioned options, an estimate of the weight of the respective protein monolayer with full surface coverage was performed by projecting the dimensions of each protein onto a surface (Table 2). The calculations were based on the largest projection area that the respective molecule may have and considered a planar surface and a maximum 2D surface packing of 91% (as given for circles; it will be lower for the proteins with an elliptical footprint depending on the aspect ratio [18]). This estimate does not include, e.g., conformational changes upon protein binding which may lead, e.g., to larger space requirements, or the effect of particle size/curvature on protein–material or protein–protein interaction during adsorption. Despite these restrictions of the model and considering also the experimental precision of protein quantification, the values exemplarily presented in Table 2 for P(MMA-Sty)67 (as the particles with the highest weight-based protein binding) suggest that the quantities of adsorbed proteins were in the range of confluent single-layer surface coverage.

For another protein, fibrinogen, the depletion from the supernatant was often >90% of the initial standardized concentration used as protein medium, thereby not corresponding to an equilibrated state and thus not being presented here in detail. Still, it should be noted that, for this more rod-like protein, it appeared that there may be a trend for an end-on rather than side-on orientation, i.e., the largest protein axis is located perpendicular to the nanoparticle surface, as suggested for other materials before [19].

The analytical methodology of protein quantification by depletion from the medium would be disturbed if non-adsorbed proteins precipitate and are removed along with the nanoparticles prior to protein quantification. However, there was no indication of such phenomena as the data analysis also suggested a monomolecular adsorbed layer. Still, for future use of such particles, e.g., in bioanalytics, a further investigation of potential structural changes of the non-adsorbed proteins is advised.

**Table 2:** Properties of employed model proteins and estimate of the space requirements for nanoparticle surface coverage by proteins compared to experimental data of P(MMA-Sty)67.

Protein	MW	Dimensions ^a^	Estimated Area Side On ^b^	Estimated Monolayer Weight Side On ^c^	Experiment P(MMA-Sty)67 ^d^
	(kDa)	(nm)	(nm^2^)	(µg·mg^−1^) (Protein/Particles)	(µg·mg^−1^) (Protein/Particles)
Bovine serum albumin (BSA)	66	9.5 × 5 × 5	48	20	19.5 ± 3.2
Immunoglobulin G (IgG)	150	14.5 × 8.5 × 4	123	18	15.3 ± 3.9
Fibronectin (FN)	450	120 × 2.5 × 2.5	300	22	45.0 ± 1.4

^a^ Dimensions as reported for albumin [20], IgG [21] and FN [22,23]. ^b^ Estimate of projection area. This estimate does not consider protein unfolding or any other reorientation; side-on orientation was here applied, meaning that the largest protein surface/axis contacts the substrate/nanoparticle. ^c^ Estimate of mass protein per mass nanoparticles in case a protein monolayer is formed. Data for particles with a diameter of 550 nm, as is the case for P(MMA-Sty)67. ^d^ Exemplary experimental data for P(MMA-Sty)67.

Considering the composition of the nanoparticles, which were prepared without the addition of steric stabilizers or polyelectrolyte brushes at their surface, the nanoparticles present a relatively smooth surface without major dangling chains. In addition to van der Waals forces, electrostatic interactions and hydrophobic interactions may be the main contributors for protein binding to these particles, probably with mixed contributions of these driving forces. Based on the similarly negative zeta potential of the particles (Figure 1B) and the negative or neutral net charge of the proteins at pH 7.4 (isoelectric point pI for BSA ~ 5, IgG ~ 7.8 [24], FN ~ 5.6 [25]), the adsorption pattern with a high surface coverage may not be explained by simply relying on net charges. It should be noted that the employed proteins exhibit distinct positively charged patches on their surface that may, in principle, contribute to a charge-driven binding of overall negatively charged proteins to negatively charged nanoparticle surfaces. Such dipole electrostatic forces may particularly account for short-term interactions. As reported, e.g., from electrokinetic binding studies of albumin, at least on a negatively charged, flat, inorganic material, proteins cannot be treated like objects with uniform charge distribution to explain adsorption behavior but instead require 3D charge distribution models [20]. However, it was beyond the scope of this study to explore potential contributions of charged patches to particle–protein interaction, as may be conducted in silico [1].

Irrespective of electrostatic interactions and as hypothesized initially, the alteration of copolymer composition with MMA as the more hydrophilic and Sty as the more hydrophobic comonomer appeared to enhance protein deposition along with increasing Sty content (increasing hydrophobicity). However, this would require sufficient distance between charged moieties on the nanoparticle surface to allow hydrophobic interaction to take place. The surface charge density *σ* can be calculated from the zeta potential *ζ* and particle radius *a* according to Equation (1) [26]:

(1) $$\sigma =\frac{2\varepsilon_{r}\varepsilon_{0}\kappa kT}{ze}sinh\left(\frac{ze\zeta }{2kT}\right){\left[1+\frac{1}{\kappa a}\frac{2}{{cosh}^{2}\left(\frac{ze\zeta }{4kT}\right)}+\frac{1}{{\left(\kappa a\right)}^{2}}\frac{8ln\left[cosh\left(\frac{ze\zeta }{4kT}\right)\right]}{{sinh}^{2}\left(\frac{ze\zeta }{2kT}\right)}\right]}^{1/2}\;\mathrm{with}\;\kappa ={\left(\frac{2IN_{A}e^{2}}{\varepsilon_{r}\varepsilon_{o}kT}\right)}^{1/2}\;I=\frac{1}{2}\sum_{i}c_{i}z_{i}^{2}$$

where *κ* is the Debye–Hückel parameter, *N_A_* is the Avogadro number, *e* is the elementary charge, *ε*_0_ is permittivity of vacuum, *ε_r_* is the dielectric constant of the medium (78 for water at 298 K), *k* is the Boltzmann constant and *T* is the absolute temperature at which the experiment was conducted (298 K),

where *I* is the ionic strength of the electrolyte medium (NaCl: conductivity 50 µs·cm^−1^), *c_i_* is the molar concentration of the respective ion *i* (4·10^−4^ mol·l^−1^,) and *z_i_* is the charge number of the ion.

Based on this, e.g., for P(MMA-Sty)67, σ = −0.00278 C·m^−2^ = −0.0174 e^−^·nm^2^ can be determined, which means that one negatively charged moiety per ~58 nm^2^ is found on average on the nanoparticle surface. This value is lower than that determined in other studies with polystyrene-based particles, where σ values of between −0.05 and −0.08 e^−^·nm^2^ were reported [27]. Considering the space requirement of sulfate headgroups such as from sodium dodecyl sulfate, which have a projection area of only 0.62 nm^2^ in water (less in electrolyte solution) [28], it may be concluded that substantial portions of the nanoparticle surfaces may indeed be available for hydrophobic interactions.

## 3. Discussion

This study analyzed the physicochemical properties of negatively charged poly(methyl methacrylate-*co*-styrene) nanoparticles in a size range of typically 550 nm and explored this set of materials as models for analyzing protein–material interaction. It was illustrated that the alteration of bulk polymer composition can have a clear impact on protein binding, as shown with three different single proteins of different biological function and molecular weights (Figure 2). With higher Sty content, the number of adsorbed proteins increased. Furthermore, higher levels of surface-area-normalized binding of FN (0.35 to 0.7 µg·cm^−2^) were observed compared to those of BSA and IgG (0.1 to 0.4 µg·cm^−2^) depending on nanoparticle composition. A parameter that could have contributed to the observed higher protein binding with increased Sty content is the higher hydrophobicity of Sty with its aromatic moiety compared to aliphatic MMA. The latter is not able to undergo π–π stacking, which is known to be the strongest interaction of amino acids side chains with nanoparticles surfaces [29]. Furthermore, charge interactions can also play a relevant role for protein binding in principle but did not appear to be dominant here since the proteins had a neutral or negative net charge, the nanoparticle surfaces were bearing negative charges as well (sulfate groups) and the surface coverage with sulfate moieties was rather low. Other relevant parameters for protein adsorption can be porosity or surface roughness. Both of them increase the accessible surface areas for adsorption, while roughness additionally can alter the protein orientation at the surface, particularly for anisotropic proteins like FN [30]. In this study, SEM analysis was used to verify the size measurements conducted by DLS using a table-top SEM with limited resolution, which does not provide details of surface structures. In other sets of P(MMA-Sty) nanoparticles synthesized under comparable conditions, high-resolution SEM revealed the absence of pores and no visual differences in surface topology of particles with increasing Sty content. Thus, possible effects of porosity or different surface roughness on protein adsorption patterns do not seem to apply for the nanoparticles reported here. Overall, despite this study not including a more comprehensive analysis of binding kinetics due to restrictions in material quantities available for screening purposes from high-throughput synthesis, the data suggest that hydrophobic interactions may play a significant role in the binding of proteins with a negative or neutral net charge to the nanoparticles, which also exhibit negatively charges.

As previously reported [3], another series of biostable copolymer nanoparticles from poly[acrylonitrile-*co*-(*N*-vinyl pyrrolidone)] (P(AN-NVP)) showed an adsorption of 0.25 to 0.7 µg·cm^−2^ for BSA and 0.05 to 0.5 µg·cm^−2^ for FN upon increasing material hydrophobicity (acrylonitrile content up to 100%) when high concentrations of protein solutions (BSA: 40 mg·ml^−1^; FN: 400 µg·ml^−1^) were used. For these P(AN-NVP) nanoparticles, at higher protein concentrations, BSA apparently showed a higher, i.e., multilayer, adsorption. However, when employing a standard concentration of 50 µg·ml^−1^, as performed here, only 0.01 to 0.06 µg·cm^−2^ for both BSA and FN was adsorbed to P(AN-NVP) (unpublished data), i.e., concentrations lower than observed here for P(MMA-Sty). This suggests that the P(MMA-Sty) series of nanoparticles may be suited to expanding the range of protein binding established by previous series of model copolymer nanoparticles.

Based on the observation that the level of bound protein systematically expanded the range of surface-bound protein quantities of P(AN-NVP), this work, beyond being a starting point for further mechanistic studies such as those on competitive protein adsorption in serum, may also contribute to establishing interfaces with a targeted level of protein retention and/or exposure to a technical or biological readout system, e.g., in bioanalytics.

## 4. Materials and Methods

### 4.1. Materials

The monomers methyl methacrylate (MMA, ≥99%, Fluka/Merck, Darmstadt, Germany) and styrene (Sty, ≥99%) were delivered by Sigma-Aldrich (Steinheim, Germany), ammonium bicarbonate solution (ABC, >99%; 6 mM) by Bernd Kraft (Duisburg, Germany) and ammonium persulfate (APS, >98%) by Merck (Darmstadt, Germany). For dialysis, Corning^®^ Costar^®^ Spin-X^®^ polypropylene centrifuge tube filters (0.22 µm pore size nylon membrane; Sigma-Aldrich) were used.

For protein adsorption studies, bovine serum albumin (BSA, A-9647, Sigma-Aldrich), fibronectin from bovine plasma (FN, F-4759, Sigma-Aldrich) and human immunoglobulin G (IgG, I-2511, Sigma-Aldrich) were employed using phosphate-buffered saline as medium (PBS, pH 7.4). All proteins were used as received.

### 4.2. Synthesis of P(MMA-Sty) Nanoparticles

The synthesis of P(MMA-Sty) was conducted in an automated parallel synthesizer platform Accelerator SLTII/106 (Chemspeed Technologies, Augst, Switzerland) as described earlier [14]. Briefly, the synthesis was conducted in ABC with APC as initiator and a total concentration of 1.57 mmol∙mL^−1^ MMA and Sty at different ratios (0:100, 25:75, 50:50, 75:25, 100:0). The samples were extensively dialyzed against water for purification using 0.22 µm membrane tubes. The number of particles in the aqueous suspension was determined by freeze-drying 10 µL particle latex in a 1.5 mL sample tube (sample freezing at −29 °C for 2 h; lyophilization using Alpha 2-4 LSC, Martin Christ, Osterode, Germany; vacuum 0.08 mbar, condenser temperature −80 °C, 19 h) and determining the dry mass with a microbalance (XP26M, Mettler-Toledo, Gießen, Germany).

### 4.3. Analysis of Particle Composition, Size, Charge and Morphology

The determination of particle composition was conducted by diffuse reflectance infrared spectroscopy (DRIFT) according to [15] using a Vertex 70 spectrometer (Bruker Optik, Ettlingen, Germany).

Zeta potential of the particles was determined by laser Doppler micro-electrophoresis of diluted particle samples in water (conductivity adjusted to ~50 µS·cm^−1^) at 25 °C using a Beckman Coulter Delsa™ Nano C (Beckmann Coulter GmbH, Krefeld, Germany).

Particle sizes were analyzed by dynamic light scattering (DLS) of diluted particle samples in water at 25 °C in quartz glass cuvettes using a Malvern Zetasizer Nano ZS (Malvern Instruments, Herrenberg, Germany). In some cases, samples were sonicated in an ultrasonic bath (5 min; Transsonic 95/HL, Elma, Singen, Germany) prior to analysis.

Particle sizes were additionally assessed by scanning electron microscopy (SEM). Diluted lattices of particles were spin-coated on a silicon wafer, sputtered with gold and examined with a Phenom G2 Pro microscope (Phenom-World, Eindhoven, The Netherlands).

### 4.4. Analysis of Model Protein Adsorption

Nanoparticle suspensions (175 µL; 2 mg·mL^−1^) were mixed with 175 µL of either BSA, FN or IgG (100 µg·mL^−1^) and incubated at 37 °C on an orbital shaker (80 rpm, Certomat IS, BBraun Biotech International, Melsungen, Germany) for 24 h. The supernatant was collected after centrifugation (50,377× *g*, 2 h; Heraeus Biofuge Stratos, Thermo Scientific, Dreieich, Germany), and the remaining protein was determined by a QuantiPro™ BCA Assay kit (Sigma-Aldrich, St. Louis, MO, USA) using a calibration curve in the linear range from 1 to 70 µg protein per ml. The protein adsorption to the particles was determined by the depletion of protein from the supernatant.

The surface coverage of nanoparticles by proteins was evaluated by a simplified calculation approach that comprised three steps: (i) determining the possible number of molecules with a given projection area on a standardized flat substrate (1 cm^2^), corrected by the highest possible 2D packing of 0.91, as applicable to circles, (ii) estimating the number of protein molecules per mass (1 mg) of nanoparticles using the particle volume (π·d^3^/6), the particle surface area (π·d^2^) and the polymer density (estimated 1.13 g·cm^−3^ [31]), (iii) calculating the corresponding mass of proteins per mg nanoparticles based on the respective protein molecular weight.

### 4.5. Statistical Data Analysis

Data of independent measurements were used to calculate statistical descriptors (mean, S.D.) of the samples.

## Figures and Tables

**Table 1:** Nanoparticle properties.

Sample	Composition		Solid Content	Particle Charge	Particle Size		Surface Area
	Sty Feed	Sty Found	c	ZP ^a^	d	PDI	A
	(mol%)	(mol%)	(mg·ml^−1^)	(mV)	(nm)		(cm^2^·mg^−1^)
P(MMA-Sty)0	0	0	109 ± 18	−45 ± 1	2313 ^b,c^	0.86 ^b,c^	26 ^b,c^
P(MMA-Sty)28	25	28	64 ± 7	−47 ± 1	539	0.19	111
P(MMA-Sty)67	50	67	26 ± 7	−52 ± 1	550	0.21	109
P(MMA-Sty)88	75	88	28 ± 8	−50 ± 1	562	0.21	107
P(MMA-Sty)100	100	100	66 ± 16	−44 ± 1	1436 ^b^	0.65 ^b^	42 ^b^

^a^ Zeta potential measured at a conductivity of 50 ± 1 µS/cm. ^b^ Data after 5 min ultrasound treatment in ultrasonic bath. ^c^ Particle sizes are overestimated and surface areas are underestimated due to aggregation.

## Data Availability

Data are available from the authors upon reasonable request.
